# How the structure of the large subunit controls function in an oxygen-tolerant [NiFe]-hydrogenase

**DOI:** 10.1042/BJ20131520

**Published:** 2014-02-28

**Authors:** Lisa Bowman, Lindsey Flanagan, Paul K. Fyfe, Alison Parkin, William N. Hunter, Frank Sargent

**Affiliations:** *Division of Molecular Microbiology, College of Life Sciences, University of Dundee, Dundee DD1 5EH, Scotland, U.K.; †Department of Chemistry, University of York, Heslington, York YO10 5DD, England, U.K.; ‡Division of Biological Chemistry and Drug Discovery, College of Life Sciences, University of Dundee, Dundee DD1 5EH, Scotland, U.K.

**Keywords:** hydrogen metabolism, iron–sulphur cluster [NiFe]-hydrogenase, oxygen-tolerance, protein film electrochemistry (PFE), *Salmonella enterica*, BV, Benzyl Viologen, IMAC, immobilized metal-ion-affinity chromatography, PFE, protein film electrochemistry, scc, standard cubic cm, SHE, standard H_2_ electrode, TM, transmembrane domain

## Abstract

*Salmonella enterica* is an opportunistic pathogen that produces a [NiFe]-hydrogenase under aerobic conditions. In the present study, genetic engineering approaches were used to facilitate isolation of this enzyme, termed Hyd-5. The crystal structure was determined to a resolution of 3.2 Å and the hydro-genase was observed to comprise associated large and small subunits. The structure indicated that His^229^ from the large subunit was close to the proximal [4Fe–3S] cluster in the small subunit. In addition, His^229^ was observed to lie close to a buried glutamic acid (Glu^73^), which is conserved in oxygen-tolerant hydrogenases. His^229^ and Glu^73^ of the Hyd-5 large subunit were found to be important in both hydrogen oxidation activity and the oxygen-tolerance mechanism. Substitution of His^229^ or Glu^73^ with alanine led to a loss in the ability of Hyd-5 to oxidize hydrogen in air. Furthermore, the H229A variant was found to have lost the overpotential requirement for activity that is always observed with oxygen-tolerant [NiFe]-hydrogenases. It is possible that His^229^ has a role in stabilizing the super-oxidized form of the proximal cluster in the presence of oxygen, and it is proposed that Glu^73^could play a supporting role in fine-tuning the chemistry of His^229^ to enable this function.

## Abstract 2

A hydrogenase consists of two subunits: a large and a small subunit. In the present study, amino acids from the large subunit were found to influence a cofactor in the small subunit, such that they help to confer oxygen-tolerance to the enzyme.

## INTRODUCTION

*Salmonella enterica* serovar Typhimurium is a Gram-negative γ-Proteobacterium and an opportunistic animal pathogen. *S. enterica* is a facultative anaerobe and as such has a remarkably flexible metabolic capability. The bacterium shows optimal growth under aerobic conditions, but in the absence of O_2_ can rapidly switch to anaerobic respiration in order to utilize alternative electron acceptors [1,2]. Molecular hydrogen (H_2_) is an important respiratory electron donor for a number of pathogenic bacteria and H_2_ respiration is known to contribute to the virulence of *S. enterica* [3–5].

**Figure 1:**
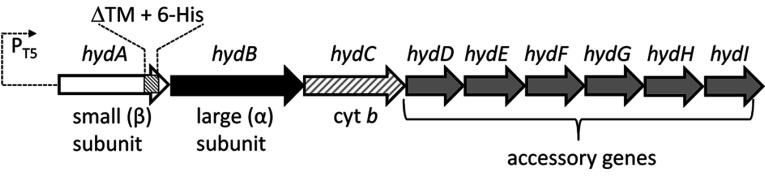
The *S. enterica hyd* operon encodes Hyd-5 The structure of the *hyd* operon (STM1539–STM1531). *S. enterica* was genetically engineered to facilitate isolation of Hyd-5. The strong T5 promoter from the pQE series of expression vectors (Qiagen) was inserted immediately upstream of *hydA* (STM1539) on the *S. enterica* chromosome. In addition, a section of the *hydA* gene encoding a TM was deleted and replaced by the sequence for a His_6_ affinity tag. The roles of specific gene products are indicated.

H_2_ oxidation (or ‘uptake’) is catalysed in *S. enterica* by three [NiFe]-hydrogenases encoded by the *hyaABCDEF* (STM1786–STM1791), *hybOABCEDFG* (STM3150–STM3143), and *hydABCDEFGHI* (STM1539–STM1531) operons. Two of these (*hya* and *hyb*) encode homologues of the Hyd-1 and Hyd-2 [NiFe]-hydrogenase enzymes found in *Escherichia coli* [6–8] and in the present study the *E. coli* nomenclature will be applied to these *S. enterica* enzymes. The third uptake isoenzyme, encoded by the *hyd* operon (Figure 1), has no equivalent in *E. coli* and its existence was revealed following assembly of the *S. enterica* genome [3,9]. The enzyme product of *hyd* has been named Hyd-5 [10]. All three *S. enterica* H_2_-uptake enzymes comprise similar ‘core’ structures with a large subunit (or α-subunit) containing the [Ni–Fe–CO–2CN^−^] catalytic centre, and a small subunit (or β-subunit) containing three Fe–S clusters. The αβ heterodimers often oligomerize into larger α_2_β_2_ heterotetramers [11–15]. The small subunits each contain single C-terminal TMs (transmembrane domains) that anchor the αβ heterodimers to the periplasmic face of the inner membrane [16]. The three *S. enterica* H_2_-uptake enzymes can therefore be subcategorized as ‘membrane-bound’ [NiFe]-hydrogenases.

*S. enterica* Hyd-1, Hyd-2 and Hyd-5 were found to be differentially expressed and utilized at different stages of infection [17]. Whereas the *hya* and *hyb* operons are up-regulated during fermentation and anaerobic respiration respectively [18,19], the *hyd* operon is optimally expressed under aerobic conditions [6]. This finding led to an initial biochemical study of Hyd-5 that revealed that Hyd-5 belongs to a class of aerobically expressed ‘O_2_-tolerant’ hydrogenases, i.e. [NiFe]-hydrogenases, that can sustain H_2_ oxidation in the presence of O_2_ [10].

The O_2_-tolerant [NiFe]-hydrogenases are of special interest with regard to their potential biotechnological applications, in particular in their utilization in platinum- and membrane-free enzymatic H_2_ fuel cells [20]. A key feature of the O_2_-tolerant enzymes is the special Fe–S cluster located proximal to the [NiFe] active site. This proximal cluster comprises an unusual [4Fe–3S] structure stabilized by six conserved cysteine residue ligands [21,22]. Unlike other Fe–S centres, this cluster is able to change conformation and reach a super-oxidized state, and it is thought that the resultant capability of releasing two electrons towards the active site is important during O_2_ attack, since it will assist in the reduction of inhibitory O_2_ to water [15,23]. As a result of this O_2_-reducing ‘rescue mechanism’, following reaction with O_2_ the O_2_-tolerant enzymes form a Ni(III) state described as the Ni-B ‘ready’ state, comprising a bridging OH^−^ between the Ni^2+^ and Fe^3+^ ions. The ‘ready’ label indicates that upon one-electron reduction of Ni(III) to Ni(II) the enzyme is reactivated rapidly back to a catalytically active form. By contrast, standard O_2_-sensitive [NiFe]-hydrogenases have a [4Fe–4S] cubane as a proximal cluster and rapidly form an inactive Ni-A ‘unready’ state upon O_2_ attack. In the absence of O_2_, all [NiFe]-hydrogenases form the Ni-B state when exposed to sufficiently oxidizing potentials.

In the present study, genetic engineering approaches have been used to overproduce *S. enterica* Hyd-5 *in situ*. A strong promoter was placed upstream of the native *hydABCDEFGHI* operon on the *S. enterica* chromosome, and a stretch of sequence encoding the small subunit TM domain was replaced with DNA encoding an affinity tag. This allowed isolation and characterization of a water-soluble active variant of Hyd-5. The crystal structure of Hyd-5 is presented at 3.2 Å resolution and reveals that a histidine residue side chain from the large subunit (His^229^) is in close proximity to the special [4Fe–3S] cluster within the small subunit. In addition, a buried glutamic acid (Glu^73^), which is peculiar to O_2_-tolerant hydrogenases, is noted within close proximity of His^229^. Electrochemical analysis of the Hyd-5 H229A and E73A variants revealed that these substitutions have dramatic effects on the catalytic properties and O_2_-tolerance of the enzyme. In the case of H229A, this includes removal of the overpotential requirement for H_2_ oxidation. The present study highlights the co-operation required between both subunits of a [NiFe]-hydrogenase in controlling reactivity of the enzyme with H_2_ and O_2_.

## MATERIALS AND METHODS

### Bacterial strains

To construct *S. enterica* strain LB03 (P_T5_, *hydA*^ΔTM−His^), DNA encoding the TM and extreme C-terminus of HydA (from the codons for Gly^314^ to Lys^367^) was deleted from the strain SFTH06 (P_T5_, *hydA*^His^) [10] as follows. DNA covering part of the *hydA* gene up to codon Gly^314^ was amplified by the oligonucleotide primers HyaATMupfor (5′-CGCGTCTAGAGAT-GTTTTGCATACTGGCTGG-3′) and HyaATMuprev (5′-CGCG-GGATCCCGTTGCCCGGCTGTAAAAAG-3′), digested with XbaI and BamHI, and cloned into similarly digested pBluescript KS+. Next, sequence encoding the HydA His_6_ affinity tag, the *hydA* stop codon, the putative ribosome-binding site for *hydB* and part of the *hydB* gene was amplified using the oligonucleotide primers HyaATMdownfor (5′-CGCGGGAT-CCAGATCTCATCACCATCACCATCAC-3′) and HyaATM-downrev (5′-GCGCGGTACCATCCCGGCGAGGATAGC-3′) and cloned into the previous pBluescript KS+ construct as a BamHI-KpnI fragment. The entire *hydA*^ΔTM−His^ allele was then excised from pBluescript KS+ using XbaI and KpnI and cloned into similarly digested pMAK705 [24]. This plasmid was used to delete the TM domain by homologous recombination as described in [24], finally generating LB03.

The derivatives of LB03 encoding the HydB E73A and H229A variants were generated as follows. DNA surrounding the Glu^73^ codon was amplified using the oligonucleotides HydBGlufor (5′-GCGCTCTAGACATGACAAATGTTACC-3′) and HydBGlurev (5′-GCGCGGTACCTTCGTCAAGGTTGATGG-3′), whereas DNA surrounding the His^229^ codon was amplified using the oligonucleotides HydBHisfor (5′-GCGCTCTAGAAAGGTCG-CGATCC-3′) and HydBHisrev (5′-GCGCGGTACCTAGC-GGACTCATCC-3′). The resulting PCR products were digested with XbaI and KpnI and cloned independently into similarly digested pBluescript KS+. QuikChange™ site-directed muta-genesis using the oligonucleotides QCHydBE73Afor (5′-GGGC-ATTCGTTGCGCGCATTTGCGGC-3′) and QCHydBE73Arev (5′-GCCGCAAATGCGCGCAACGAATGCCC-3′) for E73A, and QCHydBH229Afor (5′-GGTAAAAACCCGGCGCCTAA-CTGGCTG-3′) and QCHydBH229Arev (5′-CAGCCAGTT-AGGCGCCGGGTTTTTACC-3′) for H229A, was then performed. The substitution containing DNA fragments were excised as XbaI-KpnI fragments, cloned into pMAK705 and moved on to the chromosome of LB03 to generate the strains LB03 E73A and LB03 H229A.

### Hydrogenase assays

For whole-cell hydrogenase activity assays, Duran bottles containing 500 ml of low-salt (5 g/l) LB medium were incubated for 16 h anaerobically, without agitation, at 37°C. Cells were harvested, washed and resuspended in 50 mM Tris/HCl (pH 7.5) before BV (Benzyl Viologen)-dependent H_2_ oxidation activity was assayed as described in [25].

### Rocket immunoelectrophoresis

A Hyd-5 antiserum was raised in rabbits against enzyme purified from SFTH06 [10] and was commercially produced by Eurogentec. Duran bottles containing 500 ml of low-salt LB medium were inoculated with 0.5 ml of pre-culture and incubated for 16 h anaerobically, without agitation, at 37°C. Cells were harvested, washed and resuspended in a 50 mM Tris/HCl (pH 7.5) containing 40% (w/v) sucrose (10 ml/g of cells). EDTA (5 mM) and lysozyme (0.6 mg/ml) were added and the suspension was incubated, without shaking, at 37°C for 30 min. The sphaeroplasts were harvested by centrifugation (17000 ***g*** for 15 min at 4°C) and the supernatant was saved as the periplasmic fraction. Protein samples (2 μl) were added to small wells of a 1% (w/v) agarose gel-containing electrophoresis buffer [20 mM sodium barbitone/HCl (pH 8.6) and 0.1% Triton X-100] and Hyd-5 antiserum (7.5 μl/3 ml of gel). Samples were electrophoresed at 2 mA per plate for 16 h at 4°C. Plates were then removed, immersed in 50 mM Tris/HCl (pH 7.5) buffer containing BV and Tetrazolium Red, and incubated under an atmosphere of 100% H_2_ for 16 h. Hyd-5 activity was detected as intense red precipitin arcs.

### Preparation of proteins

Chromosomally encoded Hyd-5^ΔTM−His^ was purified from the *S. enterica* LB03 (P_T5_, *hydA*^ΔTM−His^) strain that had been cultured anaerobically in 10 litres of LB (low-salt) medium with no other additives. After inoculation with a 5 ml pre-culture, two 5 litre Duran bottles were incubated for 16 h without shaking at 37°C. Cells were harvested, washed and resuspended to a final concentration of 10 ml/g of cells in 50 mM Tris/HCl (pH 7.5), 150 mM NaCl and 75 mM imidazole (buffer A). Next, cells were lysed under pressure using the Emulsiflex-C3 homogenizer and cell debris was removed by centrifugation at 17400 ***g*** for 15 min at 4°C. The supernatant was loaded directly on to a 5 ml HisTrap HP column (GE Healthcare) equilibrated previously with buffer A. The column was washed subsequently with 15 column volumes of buffer A before bound protein was eluted by a linear gradient of 75–500 mM imidazole in the same buffer. Fractions containing Hyd-5^ΔTM−His^ were identified following SDS/PAGE (12% gel) analysis and InstantBlue staining.

Protein concentrations were determined using the method of Lowry et al. [26], whereas SDS/PAGE was by the method of Laemmli [27] and Western immunoblotting by the method of Dunn [28].

### Crystallization

Following IMAC (immobilized metal-ion-affinity chromatography), the Hyd-5 protein was purified further by size-exclusion chromatography using a Superdex 200 26/60 column (GE Healthcare) equilibrated with 20 mM Tris/HCl (pH 7.5) and 150 mM NaCl. The sample was then dialysed into 10 mM Tris/HCl (pH 7.8) and 50 mM NaCl, then concentrated using a Vivaspin 20 (Sartorius) to 6 mg/ml. This was the stock solution for crystallization.

Hyd-5 was crystallized at 20°C by the hanging-drop vapour-diffusion method using 0.75 μl of protein solution mixed with 0.75 μl of reservoir containing 19% (w/v) PEG4000, 0.1 M Mes (pH 6) and 0.24 M lithium sulphate. Brown monoclinic blocks, with the approximate dimensions 80 μm×80 μm×40 μm, grew over 2–3 days. The crystals were prepared for data collection with cryoprotection using the mother liquor adjusted to include 5% (v/v) glycerol and then plunged into liquid N_2_. Diffraction properties were characterized in-house using a Rigaku HFM007 rotating anode X-ray generator coupled to a Saturn 944HG+ CCD (charge-coupled device) detector.

### X-ray data collection, processing, structure solution and refinement

Single-wavelength diffraction data were measured on beamline I03 of the Diamond Light Source (Harwell, U.K.) using a PILATUS 6M pixel detector. Data were indexed and integrated using XDS [29] and scaled using SCALA [30]. Molecular replacement was performed using the package MOLREP [31], and a search model of the membrane-bound [NiFe]-hydrogenase from *Ralstonia eutropha* (PDB code 3RGW) [15], which shares 67% overall sequence identity for the α-subunit and 80% overall sequence identity for the β-subunit. Three αβ dimers were identified within the asymmetric unit and they are labelled A and B for the large and small subunits, and then CD and EF respectively. The model was built using electron and difference density maps and the geometry was improved in COOT [32], before refinement in REFMAC5 [33]. Local NCS (non-crystallographic symmetry) restraints were used throughout the refinement, along with TLS (Translation–Libration–Screw-rotation) refinement [34] in the latter stages. Each cycle of refinement consisted of electron and difference density map inspection and model manipulation together with incorporation of cofactors, waters and ions, followed by REFMAC5 refinement. Owing to the modest resolution of the diffraction data, only 114 water molecules were included in the model. Several Mg^2+^ ions bound at the C-terminus were included on the basis of a strong feature on the electron density maps that was consistent with metal ions found in related structures determined at higher resolution. The model of the core heterodimers was restrained to be similar with a Cα average RMSD of just 0.08 Å for copies in the asymmetric unit, therefore only the AB combination is detailed in the following discussions. MOLPROBITY [35] was used to investigate model geometry in combination with the validation tools provided in COOT and the crystallographic statistics are presented in Table 1. Co-ordinates for the Hyd-5 crystal structure have been deposited in the PDB under accession code 4C3O.

**Table 1:** Crystallographic statistics Values in parentheses refer to the highest resolution bin of approximate width 0.1 Å. *R*_merge_=Σ*hΣi*‖(*h,i*)−<*I*(*h*)> Σ*hΣi I*(*h,i*). *R*_work_=Σ*hkl*‖*F*_o_|-|*F*_c_‖/Σ*|F*_o_*|*, where *F*_o_ is the observed structure factor and *F*_c_ the calculated structure factor. *R*_free_ is the same as *R*_work_ except calculated using the 5% of the data that are not included in any refinement calculations.

Parameter	Value
Data collection	
Space group	*I*_2_
Wavelength (Å)	0.9763
Unit cell parameters	
*a*, *b*, *c*, (Å)	115.5, 122.2, 227.8
*β* (°)	95.6
Resolution range (Å)	29.5–3.2
Unique reflections (*n*)	51975
Completeness (%)	99.6 (98.6)
*<I/*σ*(I)>*	9.7 (1.8)
Multiplicity	3.8 (3.8)
*R*_merge_ (%)	10.4 (58.1)
Refinement	
Resolution range (Å)	29.5–3.2
Number of used reflections	49122
*R*_work_, *R*_free_	15.9, 20.6
Protein atoms	19918
Waters, chlorides, magnesium, sulfates	114, 7, 3, 2
RMSD from ideal geometry	
Bond lengths (Å)	0.011
Bond angles (°)	1.523
Thermal parameters (Å^2^)	
Wilson *B*-factor	83.8
Mean *B*-factor	56.9
Subunit L, S, A, B, C, D	55.0, 56.5, 59.2, 59.6, 56.0, 59.2
FeS centres (heterodimers: LS, AB and CD)	
F4S (proximal 4Fe–3S cluster)	82.0, 82.1, 82.3
FXS (medial 3Fe–4S cluster)	52.5, 56.4, 53.1
SFW (distal 4Fe–4S cluster)	55.75, 52.79, 52.59
NFU ([NiFe] active site)	53.2, 58.5, 55.7
Magnesium, chlorides, sulfates, water	49.4, 74.9, 114, 46.6
Ramachandran plot	
Favoured, allowed, disallowed (%)	92.1, 7.6, 0.3

### PFE (protein film electrochemistry)

All PFE was performed in an anaerobic glove box (O_2_<2 p.p.m.) filled with N_2_. A gas-tight glass cell housed the three electrode configuration used. Within the water-jacketed main body of the cell, the graphite working electrode was located along with a platinum wire that acted as the counter electrode. A saturated calomel reference electrode was located in a reference side arm, connected to the main cell via a Luggin capillary. A 100 mM NaCl solution was used to fill the side arm, which remained at ambient temperature. The quoted potentials have been converted into values compared with the SHE (standard H_2_ electrode) by the addition of the correction factor 0.241 V. Mixed buffer solutions [36] were prepared with purified water and were placed inside the cell at sufficient levels to cover all electrode connections. The gases were supplied by BOC and were flowed as the correct mixtures at a constant total rate of 100 scc/min (where scc is standard cubic cm) by use of mass flow controllers (Smart-Trak; Sierra Installations) connected to the electrochemical cell.

The graphite electrode surface (electrodes manufactured in-house) was prepared for enzyme application by sanding with Norton P1200 abrasive sheets directly before 3 μl of enzyme was pipetted on to the surface and adsorbed for a period of approximately 30 s. The electrode was then inserted into the electrochemical cell. This ‘working’ electrode was rotated using an Origatrod rotator (Origalys) at >3500 rev./min to allow an adequate supply of substrate and removal of product. A CompactStat potentiostat (Ivium Technologies) and the IviumSoft program were used to control the experiment.

## RESULTS

### Isolation of *S. enterica* Hyd-5

The Hyd-5 [NiFe]-hydrogenase is anchored in the periplasm by the small subunit C-terminal helix which spans the inner membrane. In the present study, the SFTH06 (P_T5_, *hyaA*^His^) strain that overproduces active Hyd-5 [10] was modified to allow removal of the TM by truncation of HydA at Gly^314^ while maintaining incorporation of a C-terminal His_6_ tag. The resultant *S. enterica* strain was designated LB03 (P_T5_, *hydA*^ΔTM−His^).

**Figure 2:**
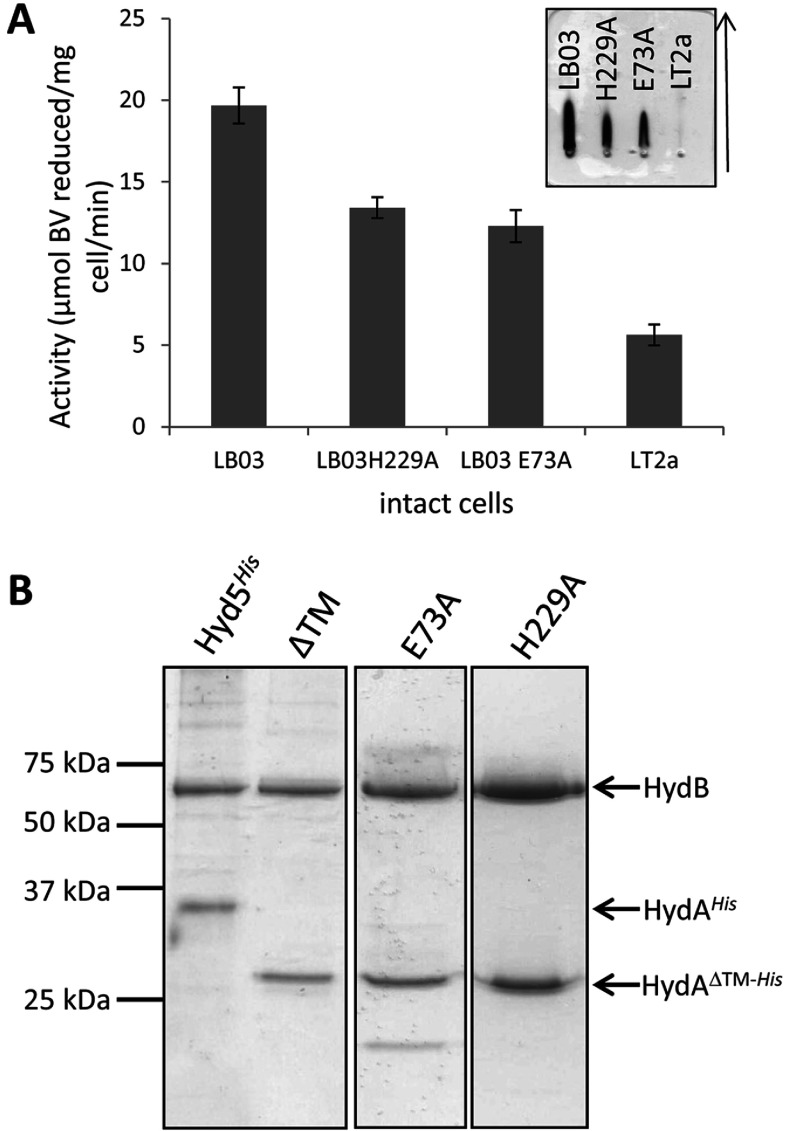
Isolation of *S. enterica* Hyd-5 and its variants (**A**) The *S. enterica* strains LT2a (parent strain), LB03 (P_T5_, *hydA*^ΔTM−His^), LB03 H229A and LB03 E73A were grown anaerobically and whole cells were assayed for hydrogen-dependent BV reduction activity. Results are means±S.E.M. (*n*=3). Inset, anti-Hyd-5 rocket immunoelectrophoresis of periplasmic fractions prepared from the strains LB03 (P_T5_, *hydA*^ΔTM−His^), LB03 H229A, LB03 E73A and the parental strain LT2a. The arrow shows the direction of protein migration. (**B**) SDS/PAGE (12% w/v gel) of pooled IMAC fractions of proteins isolated from strains SFTH06 (P_T5_, *hydA*^His^), LB03 (P_T5_, *hydA*^ΔTM−His^) (‘ΔTM’), LB03 H229A and LB03 E73A. Images taken from different gels are separated by black borders. Molecular mass is given on the left-hand side in kDa.

The LB03 (P_T5_, *hydA*^ΔTM−His^) strain was cultured at a small scale under anoxic conditions and whole cells were assayed for BV-linked hydrogenase activity (Figure 2A). The LB03 (P_T5_, *hydA*^ΔTM−His^) strain displayed increased hydrogenase activity compared with the parent strain (Figure 2A). Next, the LB03 (P_T5_, *hydA*^ΔTM−His^) strain was cultured at a larger scale (10 litres) and IMAC was employed, in the absence of detergents or anaerobic precautions, to isolate any water-soluble His-tagged proteins from a crude cell extract. Following SDS/PAGE, co-purification of the Hyd-5 large subunit (HydB, α-subunit) together with a truncated small subunit (HydA, β-subunit) was observed (Figure 2B). No other co-purifying proteins were detected and the isolated enzyme retained hydrogenase activity with BV as the artificial electron acceptor (results not shown). From this it can be concluded that up-regulation of the *S. enterica hyd* operon at its native locus, together with genetic removal of the HydA TM, results in the recovery of an active water-soluble processed and assembled [NiFe]-hydrogenase.

### The crystal structure of *S. enterica* Hyd-5

**Figure 3:**
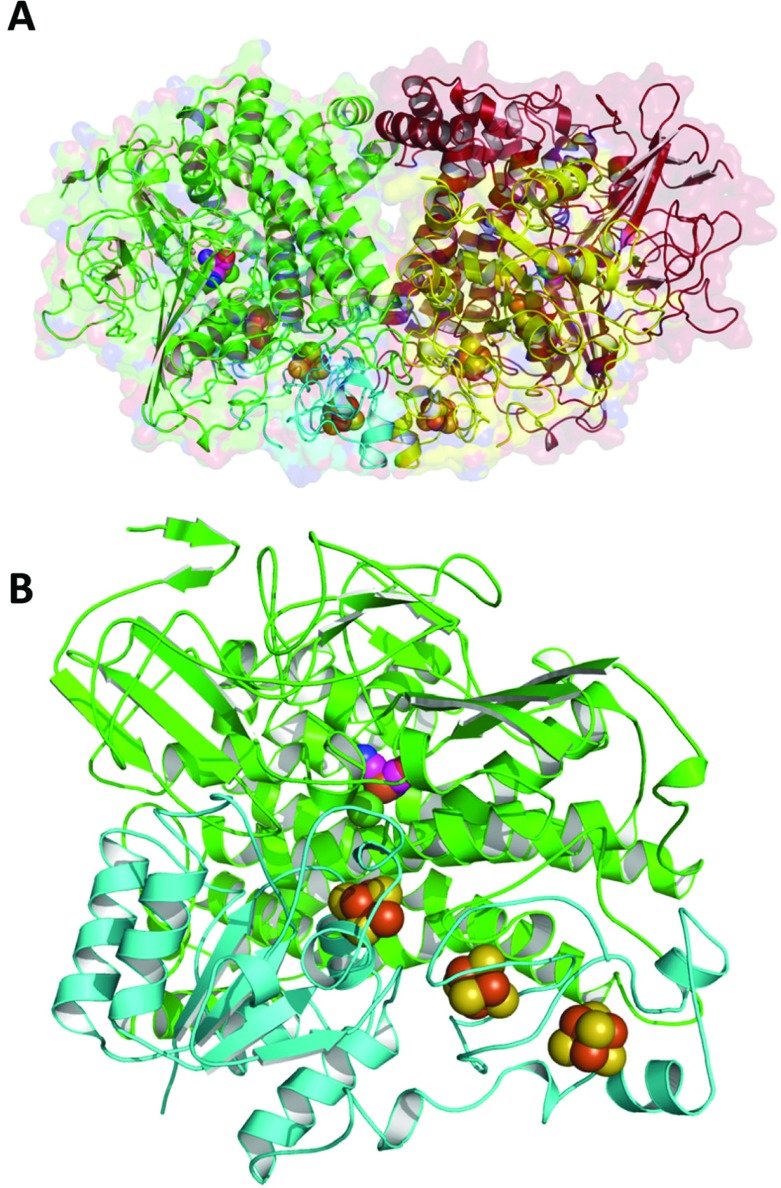
The structure of a Hyd-5 α_2_β_2_ heterotetramer (**A**) The large (α) subunits are shown as red and green ribbons, the small (β) subunits as yellow and cyan. The van der Waals surface of the protein is depicted as semi-transparent form. The spheres identify positions of the Fe–S clusters in the small subunits and the [NiFe] cluster in the large subunits. (**B**) In similar fashion, a single ‘core’ hydrogenase enzyme comprising an αβ heterodimer is shown.

The isolated Hyd-5 enzyme was subjected to size-exclusion chromatography before being concentrated and entered into crystallization experiments under standard aerobic conditions. A single crystal form was obtained that displayed space group *I*2 with unit cell lengths *a*=115.5, *b*=122.2, *c*=227.8 Å and β=95.6°. The structure was solved by molecular replacement using the structure of the membrane-bound [NiFe]-hydrogenase from *R. eutropha* (PDB code 3RGW) and refined to 3.2 Å resolution (Figure 3). Hyd-5 crystallized with three core hydrogenase αβ heterodimers in the asymmetric unit, and two of the three hydrogenase dimers associated with each other (Figure 3A) as an α_2_β_2_ heterotetramer. This α_2_β_2_ arrangement is common and had been observed for other [NiFe]-hydrogenases [10–14]. The third heterodimer in the asymmetric unit self-associates about a crystallographic 2-fold axis to produce the same α_2_β_2_ arrangement.

The C-terminus of the large subunit is well buried within the structure, which is to be expected as [NiFe]-hydrogenase large subunits are known to be C-terminally processed and locked in position before docking on to their small subunit partners [37]. In addition, available [NiFe]-hydrogenase large subunit structures have an Mg^2+^ ion that interacts with the extreme terminal histidine residue. The Hyd-5 structure also has strong feature in the electron density that would correspond to a hydrated Mg^2+^ ion.

Each Hyd-5 core heterodimer was observed to contain at least four metal cofactors at 8–11 Å separation from each other (Figure 3B). The Fe–S clusters are typical of an O_2_-tolerant hydrogenase, with a medial [3Fe–4S] cluster co-ordinated by three cysteine residues, and a distal cubane [4Fe–4S] cluster co-ordinated by three cysteines and one histidine. In the α_2_β_2_ assembly (Figure 3A), the two distal [4Fe–4S] clusters from the two β-subunits (small subunits) lie in close proximity, less than 15 Å apart (Figure 3A).

**Figure 4:**
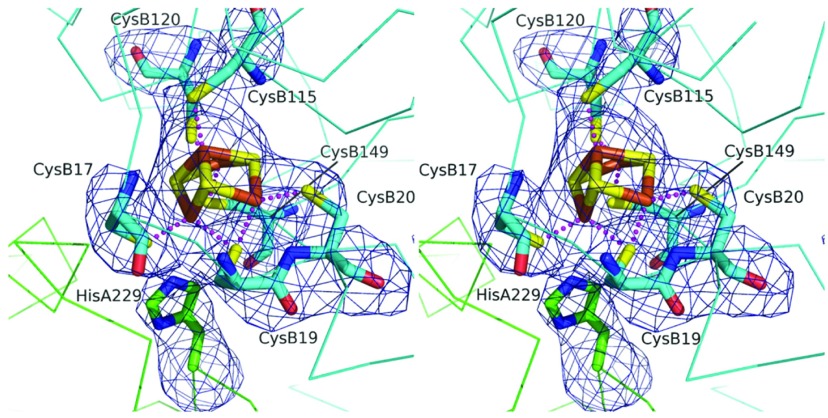
The large subunit His^229^ is close to the proximal cluster of the small subunit Stereoview of the [4Fe–3S] proximal cluster in Hyd-5. The associated cysteine residue ligands, and His^229^, are shown with the omit difference density map. The residues marked A originate in the α-subunit (large subunit), and those marked B originate in the β-subunit (small subunit). The map (blue chicken wire) is contoured at the 4σ level. The S positions are coloured yellow, Fe^3+^ brown, N blue and O red. The C positions of the small subunit are cyan and the large subunit green. Broken lines represent co-ordination links between S and Fe^3+^.

The special proximal [4Fe–3S] cluster of the small subunit is co-ordinated by six cysteine residues (Figure 4). In this structure, the backbone amide of the small subunit Cys^20^ is approximately 3.0 Å from Fe and at this resolution it is impossible to claim this provides a ligand for the metal ion, as has been suggested in alternative studies [14]. Indeed, the placement of a nearby glutamate (Glu^76^ in the small subunit), with which there is a hydrogen bond, would argue against that. Strikingly, however, a conserved histidine residue (His^229^) of the large subunit comes close to, and points directly at, the proximal [4Fe–3S] cluster (Figure 4). The distance between the large subunit His^229^ NE2 and Fe of the cluster is 3.3–3.5 Å, which is not a direct co-ordination of the metal, but certainly close enough to potentially have an effect on the properties of the cofactor (Figure 4).

### The roles of large subunit residues His^229^ and Glu^73^ in the catalytic cycle

**Figure 5:**
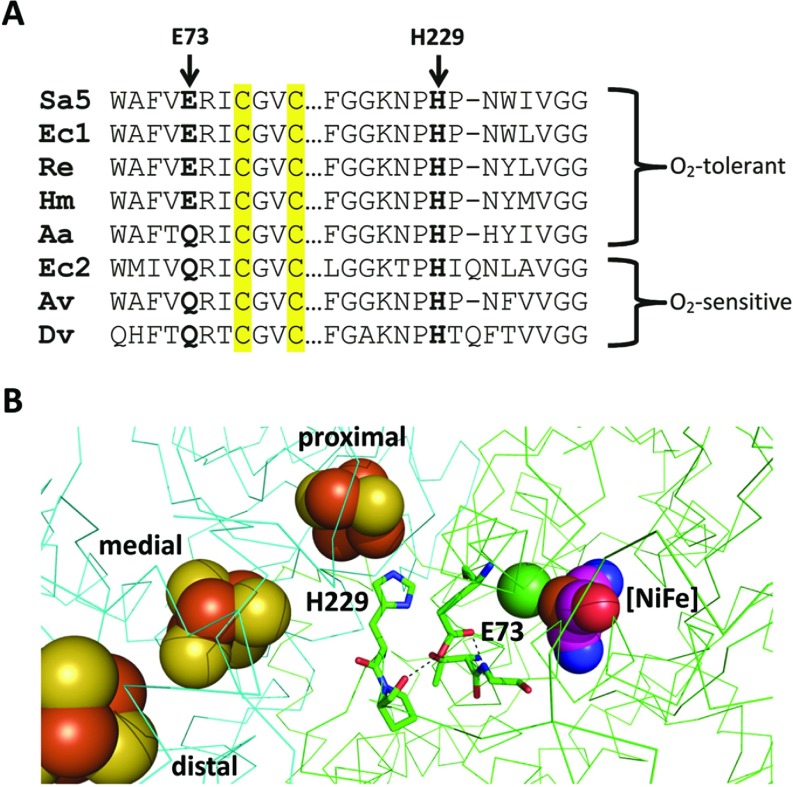
Key residues in the large subunit of O_2_-tolerant [NiFe]-hydrogenases (**A**) A sequence alignment of sections of large subunit sequences. The sequences of O_2_-tolerant hydrogenases (Sa5, *S. enterica* Hyd-5; Ec1, *E. coli* Hyd-1; Re, *R. eutropha* MBH (membrane-bound hydrogenase); Hm, *Hydrogenovibrio marinus* MBH; and Aa, *Aquifex aeolicus*) are compared with sequences from standard O_2_-sensitive hydrogenases (Ec2, *E. coli* Hyd-2; Av, *Allochromatium vinosum*; and Dv, *Desulfovibrio vulgaris*) showing the relative location of the His^229^ and Glu^73^ equivalents. Cysteine residues located in the [NiFe] active site are highlighted in yellow. (**B**) Relative location of His^229^ and Glu^73^ to each other and the four metal cofactors within the ‘resting’ structure of *S. enterica* Hyd-5. Atoms associated with cofactors are depicted as spheres and coloured according to type: S, yellow; Fe^3+^, brown; Ni^2+^, green; O, red; C, purple; and N, blue. The backbone of the β-subunit (small subunit) is traced in cyan, whereas the backbone of the α-subunit (large subunit) is traced in green. The broken lines represent hydrogen bonding between protonated Glu^73^ and the backbone carbonyl of Pro^230^ and the backbone amide of Gly^81^.

The large subunit His^229^ is completely conserved in all [NiFe]-hydrogenases (Figure 5A). In addition, the structure of Hyd-5 indicates that a buried glutamic acid (Glu^73^ of the large subunit) is close to His^229^ (Figure 5B). The Glu^73^ residue is highly conserved in O_2_-tolerant [NiFe]-hydrogenases and a glutamine residue is most commonly found at this position in standard O_2_-sensitive hydrogenases (Figure 5A). In the Hyd-5 structure, the Glu^73^ side chain appears to donate a hydrogen bond to the carbonyl of Pro^230^ in the large subunit, and this indicates that it is protonated.

In order to assess whether His^229^ or Glu^73^ of the Hyd-5 large subunit have important roles in hydrogenase activity in general, it was decided to genetically remove these side chains and replace them with alanine. The *S. enterica* LB03 (P_T5_, *hydA*^ΔTM−His^) strain was modified to yield two new strains, LB03 H229A (P_T5_, *hydA*^ΔTM−His^, *hydB* H229A) and LB03 E73A (P_T5_, *hydA*^ΔTM−His^, *hydB* E73A). To ascertain whether the variant enzymes were capable of H_2_-oxidation activity, the LB03 H229A and LB03 E73A strains were cultured anaerobically and the H_2_-oxidizing activity of whole cell samples assessed using the artificial electron acceptor BV (Figure 2A). The strains retained good levels of H_2_-dependent BV reduction activity (Figure 2A). In addition, periplasmic fractions were prepared from the same strains and samples analysed by rocket immunoelectrophoresis followed by activity staining using BV and Tetrazolium Red under H_2_-saturated conditions (Figure 2A). This technique reports on the relative levels of enzyme in different samples and also gives a non-quantitative indication of enzyme activity. No Hyd-5 activity arcs were observed in the periplasm of the wild-type strain LT2 (Figure 2A), which has no soluble periplasmic hydrogenases. However, data for the LB03 H229A and LB03 E73A mutants suggest that these enzyme variants are active and aqueous-soluble in the periplasm, albeit at slightly less abundance than the enzyme from their parent strain LB03 (Figure 2A). The Hyd-5 H229A and LB03 E73A variants were purified by IMAC and analysed by SDS/PAGE (Figure 2B). Both variant enzymes appeared physically stable with little contaminating proteins observed (Figure 2B).

**Figure 6:**
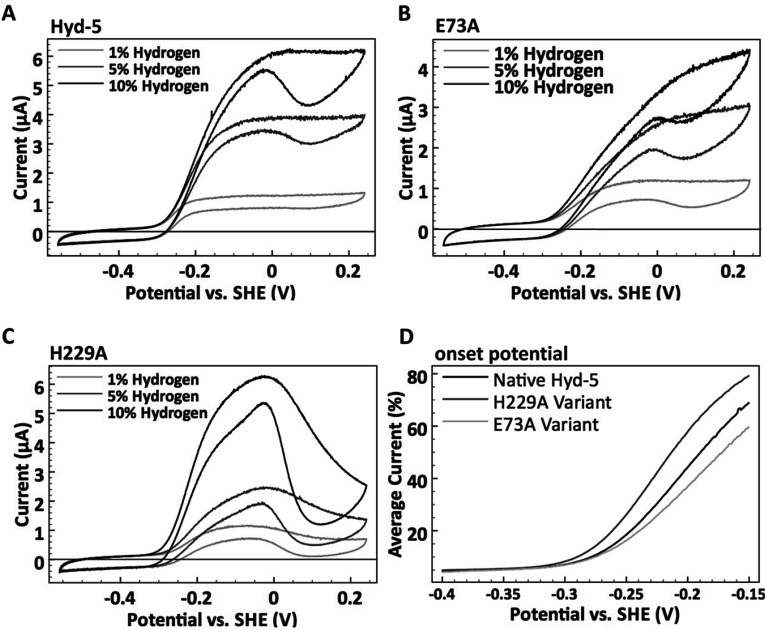
H_2_ oxidation electrocatalysis Comparing the catalytic response to H_2_ of (**A**) native *S. enterica* Hyd-5 and the (**B**) E73A and (**C**) H229A variants at pH 6.0, 37°C. Cyclic voltammograms were performed under different percentages of H_2_ as indicated. A N_2_ carrier gas was used to give a total gas mixture flow rate of 100 scc/min. The potential was increased from −0.56 to +0.24 V compared with the SHE, and then the scan direction was reversed all at a rate of 5 mV/s. The electrode was rotated at 4000 rev./min in all experiments. (**D**) To emphasize differences in catalytic onset potentials the voltammograms measured at 10% H_2_ for each enzyme are compared. The current during the forward and back potential sweep has been averaged and then normalized relative to the current at 0 V compared with the SHE.

Next, purified water-soluble native Hyd-5^ΔTM−His^, and the H229A and E73A variants, were applied separately to graphite electrodes and analysed by PFE. Figure 6 shows cyclic voltammograms of the three enzymes under differing H_2_ concentrations. At all of the percentages of H_2_ studied the response of the native Hyd-5 enzyme and the E73A variant were concurrent, with onset potentials (where a current increase marks the electrode potential where H_2_ oxidation begins) that were independent of H_2_ concentration and with a similar shape (accounting for differential adsorption of enzyme on to the electrode) (Figure 6). However, the Hyd-5 H229A variant shows a Nernstian shift in the H_2_ oxidation onset potential as a function of the percentage of H_2_ (−0.31 V compared with the SHE at 1% H_2_ and −0.34 V compared with the SHE at 10% H_2_, pH 6) (Figure 6). The relationship between Hyd-5 H229A onset potential and the concentration of H_2_ used in the assay indicates that this variant lacks the overpotential requirement seen for native Hyd-5, the E73A variant and many other O_2_-tolerant [NiFe]-hydrogenases [21,38]. At more positive potentials (greater than −0.02 V), there was also a significant fall in current for the H229A variant, where the native enzyme activity remains steady under these conditions (Figure 6). This decline in activity at higher potentials is indicative of anaerobic inactivation (formation of the Ni-B state) occurring more readily in the H229A variant (Figure 6).

### The roles of large subunit residues His^229^ and Glu^73^ in tolerance to O_2_ attack

**Figure 7:**
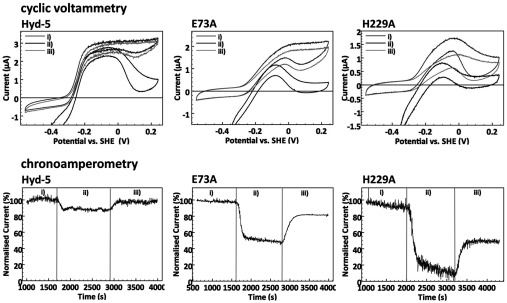
O_2_ tolerance Comparing the response to O_2_ of native Hyd-5 and the variants H229A and E73A at pH 6.0, 37°C. Cyclic voltammetry (upper panels) and chronoamperometry experiments (lower panels) compare activity before (i), during (ii) and after (iii) O_2_ exposure. Cyclic voltammetry was measured at 5 mV/s, and the potential was first swept in a positive direction, up to 0.24 V, before the scan direction was reversed. For chronoamperometry, the potential was maintained at −0.059 V compared with the SHE throughout the experiment. A N_2_ carrier gas was used to give a total gas mixture flow rate of 100 scc/min and the exact gas compositions at each stage comprised (i) 3% H_2_ only, (ii) 3% H_2_ and 3% O_2_, and (iii) 3% H_2_ (recovery stage). The electrode was rotated at 4000 rev./min.

The lack of an overpotential requirement for H_2_ oxidation activity observed in the present study for Hyd-5 H229A is reminiscent of the behaviour of native O_2_-sensitive [NiFe]-hydrogenases. Thus the effect of O_2_ exposure on the H_2_-oxidation activities of the native Hyd-5 and the H229A and E73A variants was examined. Both cyclic voltammetry and chronoamperometry experiments, which compared activity before, during and after exposure to 3% O_2_ in the presence of 3% H_2_, were employed (Figure 7). Prolonged exposure of the native Hyd-5 enzyme to 3% O_2_ had little effect on catalysis, with retention of 80% of the pre-O_2_ catalytic current at −0.059 V compared with the SHE (Figure 7), thus corroborating the O_2_-tolerance of the native enzyme [10]. Any inactivation that did occur for the native enzyme under O_2_ was probably due to formation of the Ni-B state, as the activity profiles in both the chronoamperometry and cyclic voltammetry experiments show that, following exposure to O_2_, the enzyme recovers full activity rapidly once anaerobic conditions are re-established (Figure 7). Interestingly, relative to the native Hyd-5, the application of aerobic assay conditions had a far greater effect on the catalytic activity of both the H229A and E73A variants (Figure 7). Specifically, in cyclic voltammetry the zero-current response at potentials more positive than approximately 0 V compared with the SHE shows that under these oxidizing conditions activity of the H229A and E73A variants was fully inhibited by O_2_ exposure. Additionally, in chronoamperometry at −0.059 V compared with the SHE, under prolonged exposure to 3% O_2_ the Hyd-5 H229A variant showed a continual decline in H_2_ oxidation activity, whereas at this potential the E73A variant exhibited stable activity at approximately 50% of the pre-O_2_ catalytic current (Figure 7). A proportion of the aerobic inactivation of the H229A and E73A variants was probably due to formation of the Ni-A species as full catalytic activity was never regained for either variant after O_2_ inhibition (Figure 7). Taken altogether, it can be concluded that both the H229A and the E73A variants are compromized with regards to O_2_-tolerance, although the effect of O_2_ exposure has the most negative effect in the H229A variant.

## DISCUSSION

Although the *S. enterica* genome encodes only four [NiFe]-hydrogenases, the aerobically expressed uptake hydrogenase has been termed Hyd-5 to avoid confusion with the Hyd-4 of *E. coli* [10]. Two of the three uptake hydrogenases in *S. enterica* (Hyd-1 and Hyd-5) are predicted to generate a transmembrane proton electrochemical gradient by a redox loop mechanism (similar to that suggested for respiratory formate dehydrogenases [39]) and the energy conserved by the oxidation of H_2_ is crucial for pathogenesis within the host [4]. Indeed, genetic removal of the uptake hydrogenases from *S. enterica* results in an avirulent strain incapable of invading the spleen or liver tissue [3].

Hyd-5 is of great interest as it may represent a new class of O_2_-tolerant enzyme in enteric bacteria; not only is Hyd-5 tolerant to O_2_ attack, but it is also actually expressed and assembled under aerobic conditions [38]. Understanding the molecular basis behind O_2_-tolerance, and what processes are involved in enzyme biosynthesis, are therefore important research objectives.

### His^229^ of the large subunit has an intimate relationship with the proximal cluster

In the present study, new insight into O_2_-tolerance in [NiFe]-hydrogenases has been gained. The crystal structure of *S. enterica* Hyd-5 classifies it firmly as a member of the O_2_-tolerant [NiFe] membrane-bound hydrogenases [38]. The small subunit contains a proximal [4Fe–3S] cluster co-ordinated by six cysteine residues. However, in contrast with the other available O_2_-tolerant hydrogenase structures, the *S. enterica* Hyd-5 structure reveals a large subunit histidine residue (His^229^) very close to the proximal [4Fe–3S] cluster, with a distance of approximately 3.3 Å between the His^229^ NE2 and an Fe^3+^ ion. In the [NiFe]-hydrogenase family, this histidine residue is conserved to the point of invariance, even amongst O_2_-sensitive hydrogenases (Figure 5), and was noted to be close to the proximal cluster in the first ever report of a [NiFe]-hydrogenase crystal structure [40]. The role of this conserved histidine residue in hydrogenase activity has remained largely unexplored, however, most probably because the distance between the histidine and the proximal cluster is seen to vary between structures. In the *E. coli* Hyd-1 structures, for example, the distance between the large subunit His^229^ and an Fe^3+^ of the proximal cluster is 4.2 Å, which was considered too long to be a ligand to the metal [14].

The respiratory NADH dehydrogenase (Complex I) shares an evolutionary relationship with [NiFe]-hydrogenases [41], to such an extent that Complex I contains an equivalent to the [NiFe]-hydrogenase proximal cluster called the N2 cluster, which is present in the Nqo6 subunit. Moreover, the adjacent Nqo4 subunit of Complex I has an equivalent side chain to Hyd-5 His^229^ that is close enough to the N2 cluster in Nqo6 to hydrogen bond to it [42]. The side chain is termed His^169^ in Nqo4 and Kashani-Poor et al. [43] found that when the equivalent of the Nqo4 His^169^ was mutated to alanine it resulted in the loss of the characteristic N_2_ spectroscopic signal and a concomitant reduction in enzyme activity. Furthermore, substitution of the histidine residue with methionine lowered the potential of the cluster and removed any pH dependence [44].

In the present study, substitution of the Hyd-5 large subunit His^229^ with alanine did not disrupt assembly of the enzyme, but instead had a profound effect on its catalytic properties and O_2_-tolerance, including removal of the overpotential requirement for H_2_ oxidation, which is an effect not seen in any previous studies of O_2_-tolerant hydrogenases. The native proximal cluster is known to be held at an unusually high potential in O_2_-tolerant [NiFe]-hydrogenases, and it is possible that the Hyd-5 alanine residue variant displays a lower cluster potential that leads to both the greater extent of anaerobic inactivation and loss of the H_2_ oxidation overpotential requirement observed in the present study.

The chemistry of O_2_-tolerant and O_2_-sensitive [NiFe]-hydrogenases does not only differ under aerobic conditions. Anaerobically, PFE has been used to demonstrate that both classes of hydrogenase do form the Ni-B state, but the potential at which this occurs is different for each, with O_2_-tolerant enzymes being electrochemically identifiable by formation of the inactive state at higher potentials. The O_2_-sensitive enzymes have also been shown to lack the overpotential requirement for the onset of H_2_-oxidation activity that is seen in O_2_-tolerant enzymes. Although hydrogenase variants have been described that display compromized O_2-_tolerance, to date no variant has been observed to lose the overpotential requirement for activity, which is an important problem in the search for a H_2_ production catalyst that functions in air. The loss of the overpotential requirement displayed by the *S. enterica* Hyd-5 H229A enzyme is therefore significant.

### The role of Glu^73^ of the large subunit

The presence of the large subunit His^229^ residue in all [NiFe]-hydrogenases, and Complex I, is difficult to reconcile with the clear negative effect that an His^229^ substitution has on the specialist property of O_2_ tolerance. One possible explanation is that the chemistry surrounding and influencing the His^229^ imidazole group is subtly different in O_2_-tolerant hydrogenases. The large subunit Glu^73^ is highly conserved in O_2_-tolerant hydrogenases and essentially never present in standard O_2_-sensitive enzymes, being always replaced by glutamine residue except for in the thermophilic *Aquifex* enzyme. The Glu^73^ side chain lies close to the [NiFe] active site, the His^229^ residue and the proximal [4Fe–3S] cluster. Although the Glu^73^ residue is pointing away from His^229^ in the ‘resting’ structure described in the present paper, it would only take a small conformational alteration for the side chain to adopt a different rotamer and form a hydrogen bond with His^229^. Such a conformational change would not be unusual or unreasonable to predict, and this could push His^229^ closer to the proximal cluster, or indeed pull it further away. Note that it is highly probable that His^229^ may be able to reposition itself due to the flexible region it occupies, which is rich in conserved proline and glycine residues. Interactions between glutamate/aspartate and histidine residues are very common in Nature, for example in the formation of catalytic dyad/triad charge relay systems used by some proteases [45]. It is conceivable that Hyd-5 Glu^73^ could induce or stabilize a charge-transfer network between His^229^ and the oxidized proximal Fe–S cluster in a dynamic three-way interaction. In this way, generation of a new ‘super-His’ ligand could be used to fine-tune the redox properties of the special proximal cluster.

### Conclusion

Historically, overexpression of [NiFe]-hydrogenases has been complicated by the lack of co-ordinated expression of various biosynthetic genes required for cofactor insertion and other post-translational modifications. In the present study, these issues have been largely overcome by using a genetic engineering approach to up-regulate the native operon and affinity tag a water-soluble version of the native enzyme. It was also possible to generate point mutations at the native locus and isolate fully assembled variants.

This approach has led to fresh insight into the structure and mechanism of an O_2_-tolerant [NiFe]-hydrogenase. Previous mutagenic studies have sought to understand the biochemical influence of the small subunit side chains and the Fe–S clusters, whereas studies of large subunit variants have focussed largely on the gas channel rather than the cofactor environments. Under an O_2_ atmosphere the significantly compromized O_2_ tolerance of both variants tested in the present study suggests that His^229^ may have a stabilizing interaction with the open ‘super-oxidized’ form of the proximal cluster; however, spectroscopic analyses will be required to test this hypothesis. Understanding the precise role of Glu^73^ will also require further experimentation, including production of an E73Q variant followed by further spectroscopic and enzymatic comparisons with O_2_-sensitive enzymes. It is tempting to speculate, however, that deprotonation of Glu^73^ could release it from its resting position and the resultant conformational change facilitate an interaction with nearby His^229^, which could, in turn, modulate the position of His^229^ relative to the proximal cluster during the catalytic cycle.
